# Electrolytes Disturbance and Cyclosporine Blood Levels among Kidney Transplant Recipients

**Published:** 2012-11-01

**Authors:** B. Einollahi, E. Nemati, Z. Rostami, M. Teimoori, A. R. Ghadian

**Affiliations:** *Nephrology and Urology Research Center, Baqiyatallah University of Medical Sciences, Tehran, Iran*

**Keywords:** Cyclosporine, kidney, transplantation, electrolytes

## Abstract

**Background:** Kidney transplantation is associated with various biochemical abnormalities such as changes in serum blood level of sodium (Na), potassium (K), calcium (Ca), and phosphorous (P). Although cyclosporine (CsA) is used commonly, the prevalence of its side effects, including electrolytes disturbance, is not well understood.

**Objective:** To find the prevalence of electrolytes disturbance and its relation to CsA blood levels.

**Methods:** In a retrospective study, 3308 kidney transplant recipients transplanted between 2008 and 2011 were studied. We evaluated the relation between serum Ca, P, Na, K and CsA trough (C_0_) and 2-hour post-dose (C_2_) levels.

**Results:** The mean±SD age of recipients was 37±15 years; 63% of patients were male. Overall, C_2_ levels had correlation with Ca blood level (p=0.018; OR: 1.13, 95%CI: 1.02–1.25), C_0_ levels had also correlation with blood levels of P and Cr (p<0.001; OR: 1.83, 95% CI: 1.59–2.11).

**Conclusion: **Electrolyte disturbances are prevalent. Higher serum levels of CsA can worsen the allograft function by disturbing the serum P and Ca levels.

## INTRODUCTION

Over the past two decades, cyclosporine (CsA) has been widely used as the most important immunosuppressant in kidney transplantation [1,2]. The drug, however, has several side effects including electrolyte abnormalities, *e.g.*, hyperkalemia, hypercalciuria, and hypomagnesemia [3]. It is thought that abnormal transport of electrolytes in renal tubules in transplant kidneys might be responsible for these electrolytes disturbances [4]. Some studies showed that CsA is associated with electrolyte disturbances; for example, long-term treatment with CsA increases the fractional excretion of sodium (Na) [5]. Furthermore, CsA can induce bone reabsorption and may also affect serum calcium (Ca) and phosphorus (P) concentrations [6]. In addition, hyperkalemia is frequently reported in renal transplant patients treated with CsA [7-10], which is most probably secondary to a direct effect of the drug on the distal renal tubular function and on extra-renal potassium (K) handling [11]. Moreover, CsA-induced tubular dysfunction may lead to Ca wasting, distal tubular acidosis, and hypophosphatemia [12,13]. Although kidney transplantation can improve mineral disorders, it cannot completely relieve the condition [14].

Although we could partially understand the physiopathology, molecular and genetic mechanisms of CsA-induced electrolyte disturbances [15-17], data on the prevalence of these disturbances and the role of CsA and other factors in post-transplant electrolyte disorders are scarce. We therefore conducted this study to determine the prevalence of electrolyte disturbances and its relation to CsA blood level among Iranian renal transplant recipients.

## PATIENTS AND METHODS

Between January 2008 and January 2011, 3308 kidney transplant patients from different Transplant Centers of Tehran, Iran, were retrospectively enrolled in this study. The protocol of this study was approved by the Ethics Committee of Baqiyatallah University of Medical Science.

CsA was taken orally as a basic drug for immunosuppression in kidney transplant patients; mycophenolate mofetil/azathioprine and prednisolone were also administered. To prevent a rejection, high doses of CsA were routinely started; the dosage was tapered down thereafter. The amount of the dose was initially determined by the body weight of the individuals and CsA blood Levels. Dosages also differ from one person to another depending on the patient’s ability to tolerate organ rejection. Our target for CsA trough level (C_0_) were 200–300 ng/mL during the first three months after transplantation, 100–250 ng/mL within fourth to 12 months, and 100–150 ng/mL after one year of transplantation. The 2-hour post-dose (C_2_) optimal CsA levels were 800–1000 ng/mL during the first three months after transplantation, and 400–600 ng/mL in the following months.

Routine laboratory surveys including serum creatinine (Cr), uric acid, Na, K, Ca, P, hemoglobin (Hb), cholesterol (Chol), triglyceride (TG), high density lipoprotein cholesterol (HDL), and low density lipoprotein cholesterol (LDL) were done for all patients. Blood was collected in the morning after 12-hour fast. The biochemical analyzes were done in a single laboratory using automated analyzer. All procedures for biochemical analysis had inter- and intra-assay coefficients of variation within 5%. Whole blood CsA levels were measured by Cobas Mira-Plus analyzer (Roche).

Normal levels of serum Ca and P were defined according to the recommendations of the NKF-K/DOQI guidelines regarding the stage of chronic kidney disease [18]. A plasma Na concentration of 135–145 mEq/L and a plasma K concentration of 3.5–5 mEq/L were considered “normal.”

Statistical Analysis

SPSS^®^ ver 17.0 for Windows^®^ was used for data analyses. Quantitative variables were expressed as mean±SD; qualitative variables were presented as number and percentage. Kolmogorov-Simirnov test showed that concentrations of neither CsA nor electrolytes had normal distribution; therefore, Spearman’s correlation analysis was used to study correlations between CsA blood levels and electrolytes with numeric variable such as serum Cr, age of donor and recipient. Comparison of the electrolytes with regard to sex and cytomegalovirus (CMV) contamination was performed using an independent-sample or Mann-Whitney test, considering “source of donor” as a factor. Logistic regression analysis was performed for analyzing factors with the greatest explanatory effect on electrolytes abnormal levels. A p value <0.05 was considered statistically significant.

## RESULTS

The mean±SD age of kidney recipients was 37±15 (range: 3–83) years; 63% of patients was male (Table 1). The mean±SD age of donors was 28±6 (range: 5–62) years; 83% of whom was male. The majority of grafts came from living donors (84% unrelated and 8% related), whereas 8% of patients received a deceased donor graft (Table 1).

**Table 1:** Baseline and laboratory characteristic of patients

**Variable**	**Value**
Number of patients	3308
Mean±SD age of donors (yr)	37±15
Mean±SD age of recipient age (yr)	28±6
Sex of donors (M/F), %	63/37
Donor source (DD/LRD/LURD), %	8/8/84

DD, deceased donor; LRD, living related donor; LURD, living unrelated donor

Calcium (Ca)

Of 23,154 samples that had serum Ca level, 227 (1%) were below the normal value, 14,269 (61.6%) were within the normal range, and 8658 (37.4%) were hypercalcemic. The mean±SD serum Ca level in our patients was 9.42±0.42 mg/dL.

**Figure1:**
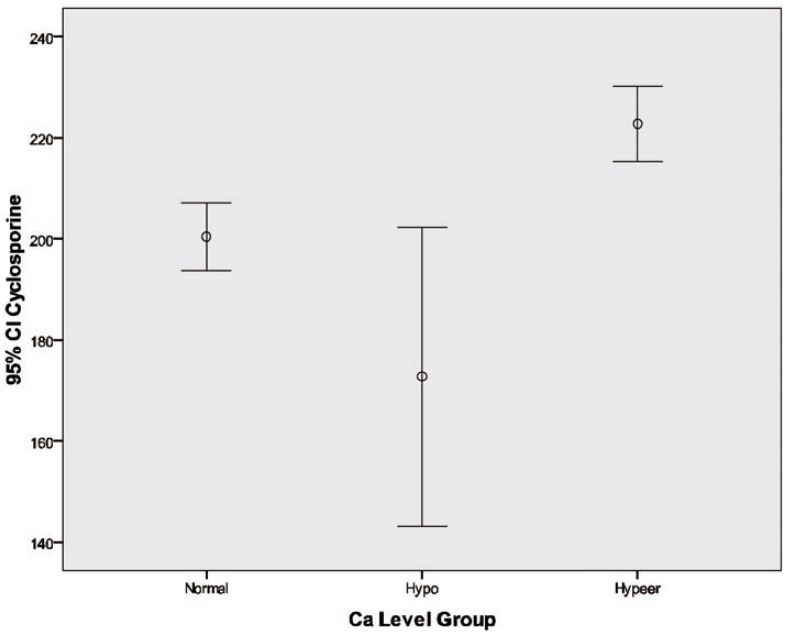
Cyclosporine blood level at different calcium level

In the multivariate model, higher serum Ca level was related to female gender, higher level of serum P, lower level of serum cholesterols and TG, and lower Na concentrations (Table 2 and Fig. 1).

**Table 2:** Factors affecting serum electrolytes

**Electrolyte**		OR	95% CI
Ca	Recipients’ gender	1.173	1.028–1.340
	P	1.570	1.428–1.728
	Cholesterols	0.997	0.995–0.998
	TG	0.999	0.998–1
	C_2_ level	1.13	1.022–1.253
	Na	0.900	0.876–0.924
P	Recipients’ gender	2.529	2.150–2.975
	Age of recipients	0.990	0.985–0.994
	C_0_ level	0.997	0.996–0.998
	Cr	1.832	1.593–2.107
	Na	1.112	1.078–1.147
	Cholesterols	0.996	0.994–0.998
	TG	1.004	1.003–1.004
	Hb	1.108	1.068–1.151
	Ca	0.517	0.434–0.616
Na	Recipients’ gender	1.482	1.195–1.837
	Age of recipients	1.008	1.001–1.014
	C_0_	0.996	0.997–1
	K	6.403	4.878–8.404
	Cholesterols	0.996	0.994–0.998
	TG	1.002	1.001–1.003
	Hb	1.071	1.018–1.126
		
K	C_0_	0.998	0.997–0.999
	Cholesterols	1.006	1.004–1.009
	TG	0.998	0.997–0.999
	Ca	1.387	1.046–1.841
	Na	1.552	1.470–1.638

Phosphorous (P)

Of 14,876 samples that had serum P level, 7398 (49.7%) were below the normal value, 7478 (50.3%) had normal P level. The mean±SD serum P level in our patients was 3.78±0.96 mg/dL.

**Figure 2:**
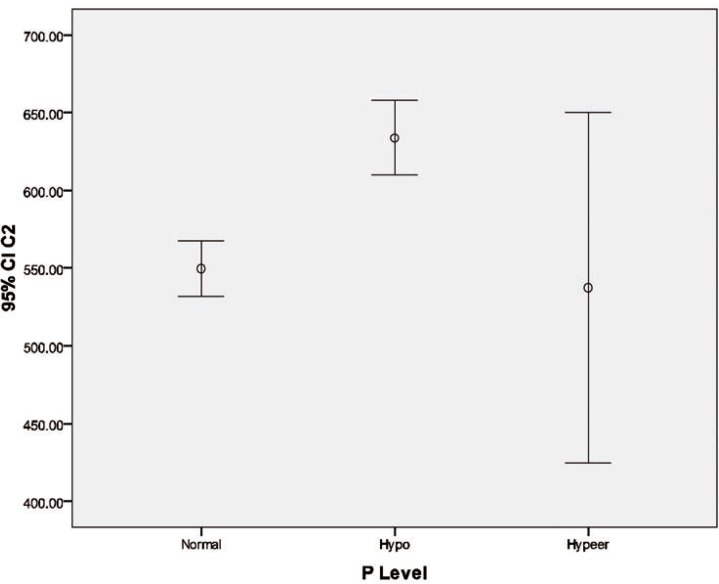
Cyclosporine blood level at different phosphorous level

Plasma level of P was linked to higher age of recipients, lower C_0_ level, higher serum creatinine, hypernatremia, higher level of cholesterols and TG, higher Hb and hypocalcemia (Table 2 and Fig. 2).

Sodium (Na)

Of 20,200 samples who had serum Na concentration, the prevalence of hyponatremia, normonatremia and hypernatremia were 16.6% (n=3344), 81.8% (n=16,251) and 1.7% (n=335), respectively. The mean±SD serum Na level in our patients was 138.2±3.0 mEq/L.

Logistic regression analysis revealed that abnormal serum Na level was correlated with female gender, higher age of recipients, lower CsA trough level, hyperkalemia, and higher cholesterols TG level (Table 2).

Potassium (K)

Among 2703 samples with serum K, the prevalence of hypokalemia, normokalemia and hyperkalemia was 5.9% (n=940), 91.2% (n=14,500) and 1.1% (n=452), respectively. The mean±SD serum K level was 4.18±0.49 mEq/L. Multivariate analysis revealed that abnormal blood K level was associated with lower C_0_, hypercholesterolemia, low TG and Ca, and hypernatremia (Table 2).

Ca, P product

Among 17,443 samples with serum Ca and P, the product more than 55 was observed in 2460 (14.1%) patients; 14,983 (85.9%) had a product less than 55.

Univariate analysis

We found significant correlation between hyponatremia, hypokalemia and hypophosphatemia with higher C_0_; we also found a significant correlation between hyponatremia and hypophosphatemia with higher level of C_2_ (Tables 3 and 4).

**Table 3:** Relation of baseline characteristic of patients with C_0_ and C_2_.

Characteristic		Potassium		Sodium		Phosphorus		Calcium	
		Mean±SD	p value	Mean±SD	p value	Mean±SD	p value	Mean±SD	p value
Donor Sex	Male	4.16±0.47	<0.001	138.42±2.88	0.09	3.71±0.90	0.45	9.36±0.42	0.000
	Female	4.23±0.50		138.51±2.66		3.65±0.89		9.44±0.45	
Recipients’ Sex	Male	4.16±0.47	0.87	138.63±2.56	0.40	3.73±0.85	0.55	9.41±0.43	0.29
	Female	4.16±0.44		138.46±2.60		3.77±0.85		9.37±0.45	
Donor Type	Deceased	4.17±0.46	0.11	138.73±2.80	0.34	3.70±0.91	0.95	9.41±0.45	0.04
	LURD	4.17±0.47		138.67±2.55		3.73±0.82		9.41±0.43	
	LRD	4.07±0.45		138.29±2.65		3.72±0.89		9.52±0.48	
CMV infection	Yes	4.22±0.52	0.63	137.92±3.10	0.52	3.73±1.01	0.051	9.36±0.44	0.14
	No	4.25±0.53		138.17±3.24		3.51±0.82		9.45±0.46	

LRD: Living related donor; LURD: Living unrelated donor

**Table 4:** Relation of serum electrolytes with C_0_ and C_2_

Electrolytes	Statistics	C_0_ blood level	C_2_ blood level	Age of recipients	Age of donors
Potassium	r	0.03	0.076	0.055	0.14
	p	0.08	0.07	0.13	<0.001
Phosphorus	r	0.14	0.104	0.112	0.029
	p	<0.001	0.016	0.003	0.49
Sodium	r	0.17	0.146	0.083	0.07
	p	<0.001	0.001	0.027	0.07
Calcium	r	0.11	0.005	0.037	0.025
	p	<0.001	0.90	0.32	0.54

Multivariate analysis

**Figure 3:**
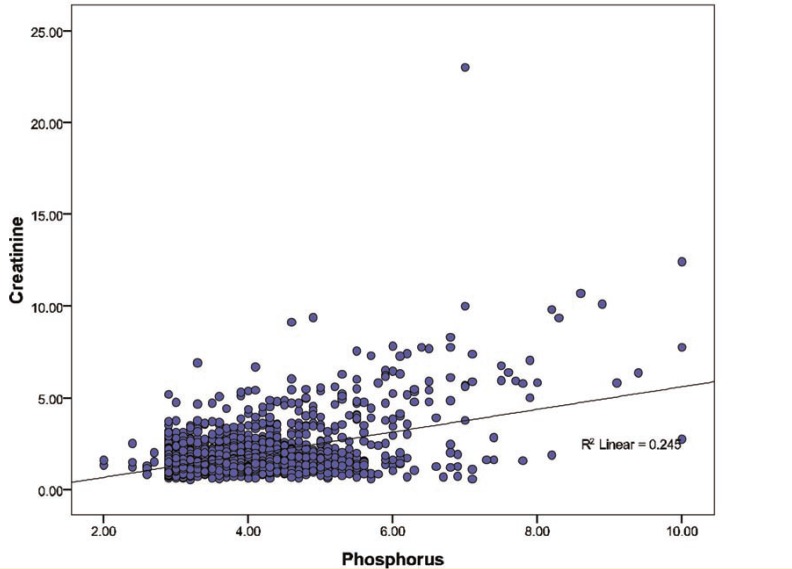
Correlation between cyclosporine blood level and allograft function

Univariate analysis showed that serum Cr concentration was significantly (p<0.001) correlated with P (r=0.495), Na (r=0.124), and K (r=0.102) levels. In multivariate analysis, among electrolytes, P had a significant association with Cr group (p<0.001; OR=1.80, 95% CI: 1.55–2.09), K (p=0.001; OR=1.53, 95% CI: 1.18–1.98), and Na had also a significant association with Cr group (p=0.031; OR=0.95, 95% CI: 0.09–0.995) (Fig. 3).

## DISCUSSION

Abnormalities in serum electrolytes especially in serum Ca and P levels are frequent in patients with chronic kidney disease and have been associated with increased morbidity and mortality [19]. Not only, has increasing evidence supported the association between abnormal electrolytes and cardiovascular risk after kidney transplantation [18], but also it showed that serum electrolytes serve as risk factors for graft dysfunction [20].

We found that half of our patients experienced hypophosphatemia and about 37% had hypocalcaemia. Derakhshan, *et al*, revealed that hyperphosphatemia and hypercalcemia were each found in 15.8%, hypophosphatemia in 8.8%, and hypocalcemia in none of their kidney transplant patients [21]. They also observed that 12.3% of their patients had a (Ca×P) product of more than 55 [21]. Moreover, hypercalcemia was observed in 66% of our kidney transplant recipients. Many factors have been proposed as the reasons; however, the persistence of secondary hyperparathyroidism, associated with a change in the set-point of Ca-controlled PTH secretion, is considered the most important factor [22]. Evenepoel showed an immediate decrease during the postoperative period; serum Ca levels increase to reach a peak after about six months [23]. In hemodialysis patients, high blood level of P is an independent predictor of mortality [24]; serum P is a predictor of mortality in renal transplant patients; it is proposed that serum P is a prognostic risk factor for kidney transplant patients [25]. Furthermore, several studies support that hypocalcaemia results in adverse outcomes and should be considered carefully [23,26].

Post-transplant hypophosphatemia caused by renal P loss is a common disorder occurring frequently after kidney transplantation due to proximal tubule dysfunction [13] which is in accord with our finding.

We observed that CsA had a significant effect on serum Na. The proximal tubular Na^+^-H^+^ exchanger (NHE3) is responsible for transcellular reabsorption of 30%–60% of the Na filtered by the glomerulus. CsA induces a reduction in absolute Na reabsorption; this effect is most probably, correlated with the decrease in NHE3 activity [15]. Moreover, under physiological conditions, the Na^+^-K^+^-2Cl^–^ cotransporter reabsorbs approximately 20% of the filtered Na^+^ and Cl. In the collecting duct, CsA may cause hypertension by stimulating the epithelial Na^+^ channel through a pathway associated with inhibition of ATP-binding cassette A1 (ABCA1) [8]. That is another evidence in favor of the importance of CsA on serum electrolytes [27].

In the present study, a positive correlation between CsA levels and serum K levels was observed; a significant correlation was found between C_0_ and hypercalemia. Hypercalcemia due to administration CsA is rare and was only reported in patients who received a renal transplant and CsA [28]. Nevertheless, a significant correlation between CsA and Na levels was observed in the present study. Our findings (excluding the inhibition of Na-K-ATPase pump due to CsA administration) suggest a hypoaldosteronism mechanism of renal toxicity that might have played a role in developing hyperkalemia and hyponatremia.

Another well-known effect of CsA is increasing the K level due to hypoaldosteronism, which has an effect on Na-K-ATPase pump. However, other studies have shown normal aldosterone levels associated with hyperkalemia in patients who received a hematopoietic stem cell transplant [29,30].

We found a significant correlation between Na, K, P and Ca with C_0_; there was also correlation between K and P with C_2_. We found a direct correlation between K and CsA, which has also been shown earlier [31,32]. Heaf showed that CsA may be osteotoxic; this might explain the negative correlation between CsA concentration and bone mass. The absence of dose-response relation does not reject this observation, since absorption f CsA is highly variable [32,33].

Our study had some limitations. Increased levels of serum P and parathyroid hormone in patients with graft dysfunction may show disturbed mineral metabolism prior to transplantation. Second, data concerning the use of phosphate binders, calcimimetics and calcium supplements did not exist between patients to investigate the comparability and the reasons behind mineral abnormalities. The duration of dialysis was significantly different among our patients. We showed in previous study that CsA absorption changes through the post-transplant period and it appears to increase over time in long-term after kidney transplantation [34]; however, we did not consider the CsA absorption effect on electrolyte disturbances.

In conclusion, electrolyte disturbances are a common problem after kidney transplantation. CsA with higher level can worsen allograft function by abnormalities in serum P and Ca levels. In addition, it seems that CsA has a strong effect on serum electrolytes post-transplantation; thus CsA effect on electrolytes should be monitored.
